# Motivational Factors Are Varying across Age Groups and Gender

**DOI:** 10.3390/ijerph19095207

**Published:** 2022-04-25

**Authors:** Hermundur Sigmundsson, Monika Haga, Magdalena Elnes, Benjamin Holen Dybendal, Fanny Hermundsdottir

**Affiliations:** 1 Department of Psychology, Norwegian University of Science and Technology, 7491 Trondheim, Norway; benjamhd@stud.ntnu.no; 2 Research Center for Education and Mindset, University of Iceland, 101 Reykjavík, Iceland; 3 Department of Teacher Education, Norwegian University of Science and Technology, 7491 Trondheim, Norway; monika.haga@ntnu.no; 4 Department of Primary and Secondary Teacher Education, OsloMet—Oslo Metropolitan University, 0167 Oslo, Norway; magdalen@oslomet.no; 5 Department of Industrial Economics and Technology Management, Norwegian University of Science and Technology, 7491 Trondheim, Norway; fanny.hermundsdottir@ntnu.no

**Keywords:** passion, grit, mindset, life-span development, cross-sectional

## Abstract

The aim of the current study was to explore differences in passion for achievement, grit, and mindset across age and gender, by using a cross-sectional design. The sample consisted of 1548 participants including 931 females and 617 males aged from 13 to 77 years (Mage 26.53 years, SD = 11.77). The eight-item Passion for Achievement Scale was used to assess general passion and the Grit-S scale was used to assess grit. Mindset was assessed using the eight-item Theories of Intelligence Scale (TIS). The results indicated significant differences between the three factors related to age, age groups, and gender. For the total sample, there was a significant gender difference in passion, where males score higher, and growth mindset, where females score higher. With age, passion decreases until the age of 50–59, and slightly increases for the remaining age groups. After a decrease in grit between the first (13–19 years) and the second (20–29 years) age group, grit increases with age. Mindset scores decline strongly after the age of 40–49. Generally, the patterns show that mindset and passion decrease across the life-span, while grit increases. Indeed, these attributes seems to be different from each other, and how they change varies across age groups.

## 1. Introduction

A way to improve insights in how individuals face challenges, pursue long-term goals, and maintain effort in school and life is to delve into the factors of growth mindset, passion, and grit. In the last few decades, there has been a growing interest towards these potential predictors to high achievement and excellence [1,2,3,4,5,6], and promising findings are found regarding their association to motivation and success [2,4,5,6,7,8,9,10]. Despite increased interest in these factors, our knowledge of their developmental patterns throughout the course of life are still scarce. Hence, seeking to explore passion, grit, and mindset through a cross-sectional design is a novel approach in discovering variations related to age and gender within these concepts.

*Passion for achievement.* Most experts justify their exceptional motivation to a strong interest or passion [11], and passion can just be defined as “a strong feeling toward a personally important value/preference that motivates intentions and behaviors to express that value/preference” [12] (p. 1). Passion is often put in relation to dedication, enthusiasm, persistence, goal orientation, liking, and even love [13]. Moreover, it is an intense affective state, which may produce beneficial effects on skill development based on its key mechanism “*immersion*” i.e., a deep mental involvement [12]. Consequently, passion may be providing the focus necessary for long-term goal achievement [9,14]. Usually, passion has been assessed in relation to specified activities, and thus been domain-specific [15]. However, a recently developed scale has operationalized passion independent from a specific activity, and focused more on achievement in general [8]. In this context, passion can represent a trait, or a tendency, to develop strong interests toward areas, themes, or skills in general. Gender differences have been found in passion for achievement, males scoring higher [7,16].

*Grit.* When striving against a long-term goal, some activities or situations may be experienced as quite challenging or even boring for an individual. Consequently, one needs persistence of effort and grit in order to stay on course. Duckworth et al. [17] define grit as “*perseverance and passion towards long-term goals*” (p. 1). Generally, grit is described as a trait that entails working diligently toward a challenging goal through thick and thin, over many years, and even decades [18]. It consists of two underlying facets: “Consistency of Interests” reflecting the stability of a person’s interests over longer periods of time, and “Perseverance of Effort” which means diligence and effort despite difficulties or failure [17]. Although grit may have similarities to resilience in terms of striving through adversity [19], (it is to a greater extent characterized by long-term goals and consistent interest) [20,21,22]. As a result, gritty people manage to develop high skills through hard work and zeal to achieve their long-term goals. Furthermore, grittier individuals tend to attain higher levels of education and earn a higher Grade Point Average [17]. However, the concept has received a lot of reasonable criticism concerning its facets, predictability, and similarity to other existing concepts such as Conscientiousness in the “big five” [23,24,25]. The “big five” are broad categories of personality traits (openness, conscientiousness, extraversion, agreeableness, and neuroticism) [26], and strong associations are found between grit and conscientiousness) [17,27,28]. In addition, grit can be an effective measure to assess proactive conscientiousness [29]. Using the original 12-item grit scale, gender differences have been found; female score higher [25,30]. When using the Grit-S scale, gender differences are not found [30].

*Mindset.* Grit has also been related to possessing a “growth mindset” [4]. Having a growth mindset means believing in the development of skills by practice and experience [31]. In contrast, people with a “fixed” mindset assume they are born with a certain amount of talent or intelligence, and that it cannot be changed [31]. Thus, growth mindset may play a key role in the motivation and achievement of an individual, and influence how a person approaches learning opportunities, challenges, and goals [31], which in turn, can influence motivation and effort. Mindset and motivation are important factors in improving math performance in high school students [32]. Park et al. [22] argue that the two attributes mindset and grit seem to mutually reinforce each other. Furthermore, people with a growth mindset have a smaller tendency to worry about learning outcomes, and invest more time and energy into learning [33]. A short online growth mindset intervention was found to improve grades among lower-achieving students [6], yet it seems that the concept has a general weak effects [34], indicating that having a growth mindset is even more effective in combination with other relevant traits. Previous findings show that females and males either do not differ in mindset on average [35] or that females hold a higher growth mindset than males [36,37].

*Passion, grit, and mindset.* Passion, grit, and mindset are intertwined constructs that carry advantages for high achievement. It can be argued that growth mindset may be an underlying factor for both passion and grit, indeed, individual’s belief about the malleability of personal attributes and abilities affects an individual’s behavior in terms of goals and actions. However, the opposite might also be true, and therefore passion, grit, and growth mindset seem to be attributes whose development are mutually reinforcing [5,7,9,22]. Most studies concerning passion, grit, and mindset have focused on each specific concept in relation to performance and motivation [6,11,18], while little is known about the development of these traits across the life-span. Although some studies have explored the associations between other factors and these traits in different age groups, such as grit and work life [38,39], (specified passion in art performance [11] and mindset and academic performance [40,41], there is limited knowledge about variations in passion, grit, and mindset, related to age and gender. Studies indicate that grit increases with age, suggesting that grit changes as the individual acquires different experiences throughout the life-span) [17,19]. However, these possible variations and changes should be further investigated to explore their significance.

The current study is part of a larger project focusing on these motivational factors, i.e., passion, grit, and mindset. Earlier studies have explored the patterns of association between these three concepts across the life-span, as related to age and gender [9,16]. In this current paper, we continue to study passion, grit, and mindset, with a special interest in the development of these three variables across the life-span in a cross-sectional sample among seven age groups from 13 to 79 years (N = 1548). Hence, this study is further exploring how these central variables are characterized in different age groups between gender and through different periods in life. As far as we know, this has not been earlier investigated and will add new understanding about these variables. Both gender- and age differences across life are probably influenced by different life experiences that affects in different ways [42]. It is therefore hypothesized that passion, grit and mindset might be independent of each other, and vary through life-span. The aim of this study is to explore age and gender differences in passion, grit, and mindset using a cross-sectional design in the age span from 13–77.

## 2. Method

### 2.1. Participants

A sample of 1548 participants from 13 to 77 years (M = 26.53, SD = 11.77), completed scales for passion, grit, and mindset (dependent variables) during 2019/2020. Participants were recruited from two Nordic countries, Norway (N = 838) and Iceland (N = 710). Mean age for the female sample (N = 931) was 26.70 years (SD = 11.80), and 26.27 years (SD = 11.71) for the male sample (N = 617). Adolescents from 13 to 19 years (N = 242) were recruited from mainstream secondary schools and high schools. The entire sample reflected the population of adolescents attending schools in these areas and included adolescents from a wide range of socio-economic backgrounds. The adults aged 20–77 years (N = 1306) were recruited from a university student population (tested at university campus in a group setting in ), sports clubs (football players, female and males at different levels), and group of visitors to a public building (tested individually). The participants were divided into seven age groups based on chronological age: 13–19, 20–29, 30–39, 40–49, 50–59, 60–69, 70–79. Participants aged 19 years and younger (high school students) as well as participants over 70 years (people with pensions in Norway) were divided in two separate groups (13–19 years and 70–79 years). The age range from 20 to 70 years was divided into five groups at 10-year intervals (a decade apart). The information registered about the participants was anonymous (except age and gender). The sample can be described as a convenience sample.

### 2.2. Measurements

#### 2.2.1. Passion

Participants completed the Passion scale [8] as a measure of passion for achievement. Participants indicated their responses to eight items on a 1 (*not like me at all)* to 5 (*very much like me)* scale. The maximum score on this scale is 5 (extremely passionate) and the lowest is 1 (not at all passionate). The Passion for achievement scale has demonstrated good internal consistency (Cronbach’s alpha value of 0.86) and high levels of test-retest reliability. Intra class correlation coefficient (ICC) between test and retest total scores was 0.92 (N = 21, mean age 23. 67, SD =2.41). Construct validity: Pearson’s correlation coefficient between the total score of the Passion and Grit S Scale was 39 for adults, mean age 21.23 (SD = 3.45) (N = 107) [8], and 0.54 for adolescents, mean age 17.85 (SD = 1.47) (N = 242) (this study). For this particular study, the Chronbach’s alpha value was 0.92, indicating a high internal consistency (N = 1548).

Principal component analysis: An exploratory principal component analysis was used in this study (N = 1548) to investigate the component structure of the 8-item passion-scale [8]. A one-component structure was extracted based on the inspection of eigenvalues, scree plot, and theoretical sensitivity [43]. The items characterized a component that was named “Passion for achievement” and had an eigenvalue of 5.23. In addition, the component explained approximately 65.48% of the variance. Factor loadings ranged from 0.73 to 0.87, which indicates good loadings with the latent component. KMO was 0.91 which indicated an adequate sample size, and the significance of the Bartlett’s test suggested that the variance was the same in each group. A good dimensionality and adequate factor structure of the passion scale has been confirmed through exploratory factor analysis and confirmatory factor analysis in a Turkish sample [44].

#### 2.2.2. Grit

Participants completed a Norwegian version of the Grit S Scale [19,45] as a measure of level of grit. The scale has two dimensions: consistency of interests (COI) (e.g., “I often set a goal but later choose to pursue a different one”) (reverse-scored), and perseverance of effort (POE) (e.g., “I finish whatever I begin”). All eight items were measured on a 5-point Likert scale, wherein 1 would mean “not like me at all” and 5 would mean “very much like me”. The maximum score on this scale is 5 (extremely gritty), and the lowest score is 1 (not at all gritty). Grit-S has shown good internal consistency in several studies, α = 0.82 and α = 0.84 [19] (p. 170), and provided evidence for the predictive validity, consensual validity, and test-retest stability of the Grit-S. For this particular study, the Chronbach’s alpha value was 0.73, indicating a good internal consistency (N = 680).

#### 2.2.3. Mindset

Participants completed a Norwegian version of Dweck’s [31] Theories of intelligence Scale (TIS) as a measure of mindset [46]. The self-form for adults of this measure was used to ensure that the students focused on their ideas about their own intelligence and not their ideas about people in general. In completing the scale, participants indicated their agreement or disagreement using a 6-point scale (1 = strongly agree to 6 = strongly disagree) on a variety of items related to the malleability and stability of intelligence and talent. The scale consists of two subscales, and the items were presented so that agreement indicated either support for an entity theory, i.e., fixed mindset (e.g., You have a certain amount of intelligence, and you can’t really do much to change it) or an incremental theory, i.e., a growth mindset (e.g., No matter who you are, you can significantly change your intelligence level). Before summing all items, the incremental scale items were reversed. Therefore, higher average scores indicate a greater amount of incremental beliefs about intelligence i.e., growth mindset. The reliability data for the scale comes from Dweck et al. [47] and is based on the 8-item scale. The scale showed good internal consistency (α = 0.85) and test-retest reliability at two weeks (*r* = 0.80). Additionally, the scale showed a good construct validity, with scores predicting a meaningful relationship with several variables [47]. The Norwegian version of TIS has been found to be reliable as well, with Cronbach’s α of 0.86 for entity items and 0.88 for the incremental items [46]. For this particular study, the Chronbach’s alpha value was 0.93. (N = 680), indicating a high internal consistency.

### 2.3. Procedure

The study was carried out in accordance with the regulations set out by the Norwegian Centre for Research Data and the Icelandic Data Protection Authority. Before data collection, participants in the adolescents group (i.e., younger than 16 years) and their parents or guardians were given written information about the study. For the adolescent group, written permission was obtained from parents or guardians before involvement in the study. According to the Norwegian Centre for Research Data and the Icelandic Data Protection Authority, passive consent was sufficient for participants older than 16 years, as no sensitive personal data were collected. Research assistants carried out data collection. The data collection was both conducted by using online survey (mainly adolescents and adults) and distribution and collection in person (the youngest and oldest groups).

### 2.4. Data Analysis

For the statistical analysis, SPSS Version 25 for Windows was used (SPSS Inc., Chicago, IL, USA). Pearson’s correlations analyses were used to analyze the relationship between age and the three factors, and *t*-test was used to analyze the difference between genders. Multivariate analyses of variance (MANOVA) were used to analyze the difference between the three factors related to age, seven age groups, and gender. To counteract the problem of multiple comparisons, the Bonferroni’s correction was used for analyzing difference between groups within each factor: passion, grit, and mindset. The magnitude of partial eta squared was determined following the thresholds: η2 = 0.01 indicates a small effect; η2 = 0.06 indicates a medium effect; η2 = 0.14 indicates a large effect. Statistical significance was set to *p* < 0.05.

## 3. Results

### 3.1. Demographic Differences

As a first step, demographic differences among variables of interest were explored. Age correlated significantly with mean total score Passion (*r* = −0.135, that is the higher the age the lower the passion score) and mean total score Grit (*r* = 0.144, i.e., higher age—higher grit score). No significant correlation was found between age and mean total score Mindset (*r* = −0.009, i.e., higher age—lower mindset) (Pearson’s correlation, *p* < 0.01). For the whole sample, the mean score for passion was 3.95, for grit 3.39, and for mindset 4.21. There was a significant difference between females and males for the average total score of passion for achievement (*t*-test, *p* < 0.001), males having a higher score. A significant gender difference was also found in total score for mindset, females having a higher score (*t*-test, *p* = 0.023). For grit, no gender difference was revealed. (see Table 1). The correlation between passion and grit was *r* = 0.330, the correlation between passion and mindset was *r* = 0.158, and the correlation between grit and mindset was *r* = 0.177. The correlation was significant (*p* < 0.01).

**Figure 1:**
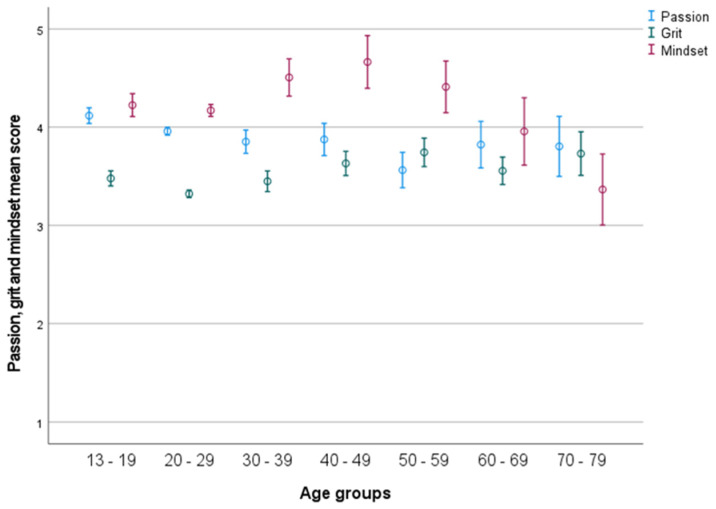
Passion, grit, and mindset across life-span in the seven age groups. Error bars represent 95 CI.

MANOVA indicated a difference between the three factors related to: (1) age (*F*(189, 4225) = 1.735, *p* < 0.001), with a medium effect size (partial η2 = 0.071); (2) age groups (*F*(18, 4274) = 9.540, *p* < 0.001), with a small effect size (partial η2 = 0.035); and (3) gender (*F*(3, 1409) = 5.875, *p* < 0.001), with a small effect size (partial η2 = 0.014) (see Figure 1).

There was a significant interaction effect between gender (2) and age (*F*(153, 4224) = 1.217, *p* = 0.038), with a small effect size (partial η2 = 0.042). There was also a significant interaction effect between gender (2) and age groups (seven age groups) (*F*(18, 4274) =1.841, *p* = 0.016, with a micro effect size (partial η2 = 0.007).

### 3.2. Passion

**Figure 2:**
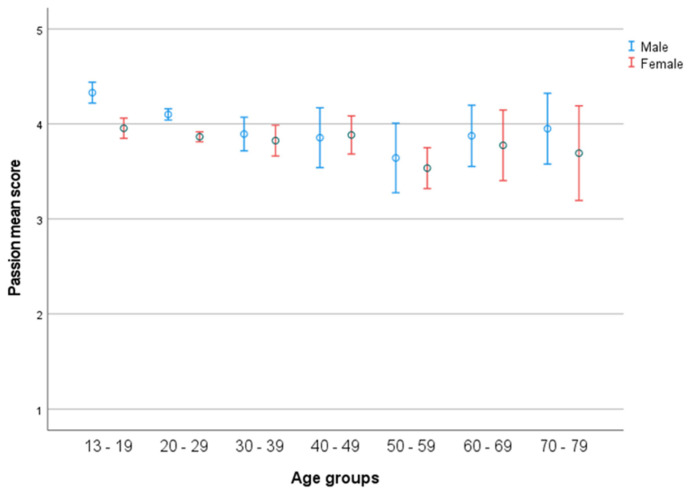
Passion across life-span in the seven age groups in relation to gender. Error bars represent 95 CI.

There was a significant effect of age (*F*(63, 1411) = 1.589, *p* = 0.003), with a medium effect size (partial η2 = 0.064), and of age groups (*F*(6, 1513) = 6.986, *p* < 0.001), with a small effect size (partial η2 = 0.026) (see Figure 1). Post hoc test indicated a significant higher score in passion for group 1 (13–19) compared to group 2 (20–29), group 3 (30–39), and group 5 (50–59). Furthermore, there was a significantly higher score in passion for group 2 than group 5. A significant effect of gender was found (*F*(1, 1513) = 6.141, *p* = 0.013) with a small effect size (partial η2 = 0.004) There was no significant interaction between gender (2) and age-groups (seven age groups) (*F*(6, 1513) = 1.364, *p* = ns) (see Figure 2), with a micro effect size (partial η2 < 0.005). *t*-test indicated significant gender differences in age group 13–19 (*p* < 0.001) and 20–29 (*p* < 0.001), males having higher scores.

### 3.3. Grit

**Figure 3:**
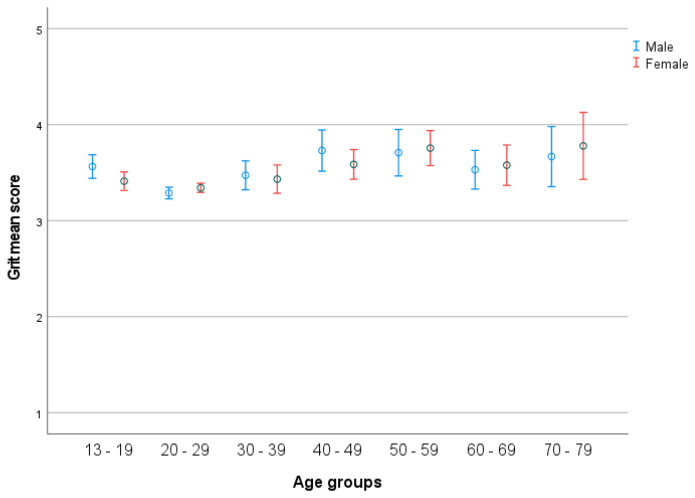
Grit across life-span in the seven age groups in relation to gender. Error bars represent 95 CI.

There was a significant effect of age (*F*(63, 1411) = 1.589, *p* = 0.003), with a medium effect size (partial η2 = 0.069), and of age groups (*F*(6, 1513) = 9.895, *p* < 0.001), with a small effect size (partial η2 = 0.037) (see Figure 1). Post hoc test indicated a significantly higher score in group 1 compared to group 2, and significantly lower than group 5 and group 7. Furthermore, there were significantly lower scores in group 2 compared to group 4, 5, and 7. Additionally, group 3 scores were significantly lower than group 5. There was no significant effect of gender (*F*(1, 1513) = 0.040, *p* = ns.) and no significant interaction between gender (2) and age groups (seven age groups) (*F*(6, 1513) =1.224, *p* = ns.) (see Figure 3) with a micro effect size (partial η2 < 0.0001). *t*-test indicated significant gender differences in age group 13–19, males having a higher score (*p* = 0.048).

### 3.4. Mindset

**Figure 4:**
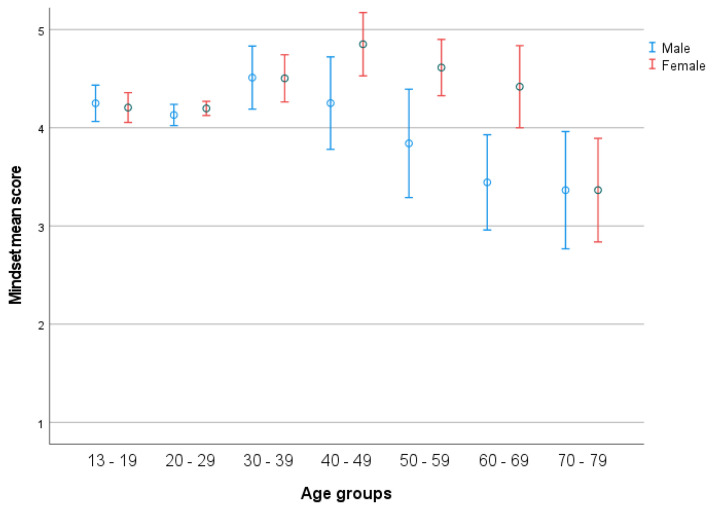
Mindset across life-span in the seven age groups in relation to gender. Error bars represent 95 CI.

There was a significant effect of age (*F*(63, 1411) = 1.540, *p* = 0.005), with a medium effect size (partial η2 = 0.069), and of age groups (*F*(6, 1513) = 9.895, *p* < 0.001), with a small effect size (partial η2 = 0.024) (see Figure 1). Post hoc test indicated a significantly higher score in group 1 compared to group 7. Additionally, group 2 had a significantly higher score in mindset compared to group 7, and group 3 scored significantly higher in mindset compared to group 7. There was a significant effect of gender (*F*(1, 1513) = 11.629, *p* < 0.001) with a micro effect size (partial η2 = 0.007) (see Figure 4), and a significant interaction between gender (2) and age groups (seven age groups) (*F*(6, 1513) = 2.886, *p* = 0.006), with a small effect size (partial η2 = 0.012). *t*-test indicated significant gender differences in age group 60–69, females having higher scores (*p* = 0.037).

## 4. Discussion

The aim of the study was to explore the significance of age and gender related differences in passion, grit, and mindset across the life-span. The three previously described scales were administered to 1548 subjects, covering females and males aged from 13 to 77 years. Previous research has argued that these factors are important for achievement) [1,2,4,5,8,9,10,48,49]. However, very little is known about the development of passion, grit, and mindset across different age groups and during aging. Seeking to explore these factors through a cross-sectional approach is a novel approach in discovering differences related to age and gender within these concepts.

The overall results show that with age, passion decreases until the age of 50–59 years, and slightly increases for the remaining age groups. After a decrease in grit between the first (13–19) and the second (20–29) age group, grit increases with age, as observed in earlier studies [17]. Mindset scores decrease clearly after the age of 40–49 years. Generally, the different development in patterns in the three concepts across the seven included age groups may support that passion, grit, and mindset can be seen as different constructs [9,50]. The only similarities in patterns are found between mindset and grit in the four youngest age groups, with a decrease from 13–19 to 20–29 years, followed by an increase. Furthermore, both passion and grit increase slightly after the age of 50–59. Generally, the patterns show that mindset and passion decrease across the life-span, while grit increases. Indeed, these attributes seems to be different from each other, and how they change varies across age groups.

### 4.1. Passion

Studying the development of passion from an ecological perspective, the individuals in the age interval of 13–19 years could be freer to interact with the microsystems including their passion [51], making their average scores high (4.11). Decrease in mean passion scores until the age of 50–59 could be a result of restraints in the environment. Often great life events occur during the ages of 20–29 and 30–39, such as studies, the start of working careers, and establishing families. This could possibly restrict the availability of microsystems that involve the individuals’ passion, and consequently, their passion could decrease due to new/great commitments to other arenas. Similarly, when passion increases in the age interval 50–59, it could be a result of children moving out, retirement, and the availability of microsystems including their passions such as hobbies and leisure activities [8]. Such changes in the average passion scores may question Swanson [52] claiming that interests stabilize over time. The analysis of gender differences revealed that on average, males tend to score higher in passion compared to females, across all age groups. This might indicate that passion is a stronger driving force for males [9,50]. In this respect, it might be argued that passion provides the focus that is important in achieving goals [4]. Passion can therefore be of special importance, and guide an individual toward a specific area of interest [8,9]. Among males, passion scores decline between the age interval of 13–19 to 50–59, followed by increases in the two oldest age groups. Females show the same trend, with a weaker decline in passion scores compared to males appearing between the age interval of 13–19 to 50–59. The scores slightly increase among the remaining, older age groups. These findings could potentially be caused by both sociocultural factors and neurobiological differences between males and females [53,54,55]. Sociocultural expectations may contribute to males becoming more passionate about the activities in which they engage [9,16]. Moreover, the availability of activities capturing males’ interests (such as gaming) could also explain the significant gender difference in passion. Generally, males show higher addictive tendencies towards not only alcohol, but also towards activities such as gambling, television, and internet use [56]. Consequently, it is tempting to speculate whether there is a relationship between passion, addiction, and the interaction between dopamine, serotonin, and sex hormones [57,58]. In this context, studies have indicated higher levels of dopamine in males compared to females [59].

### 4.2. Grit

Average grit scores are low in the age interval 14–19 year compared to older age groups. As proposed in earlier studies, this might indicate that grit is developed in interaction with various microsystems during the life course, for example by facing challenges and difficulties that could develop grit [17]. Indeed, in the interval from 20–29, the mean grit scores increase, which could be a result of individuals interacting with different microsystems, including attending to universities, and participating in work life [17].

Gender differences in grit scores are only found in the youngest group (13–19), where males score significantly higher on grit than females (3.56 vs. 3.41). Some studies report on no gender differences in grit scores, and that the trait increased with age in a sample of adults from 25 to 65 years [17]. On the contrary, others have shown that females score generally higher in grit compared to males) [30], and they were unable to detect a linear relation between grit and age, both with and without statistically controlling for the effects of education [42]. Considering that various studies contain different samples, the observed variability of the results can be caused by sociocultural factors, and grit might be adaptable.

### 4.3. Mindset

Results showed that growth mindset decreases throughout the life-span, from the age of 13–19 (mean score 4.22) to 70–79 (mean score 3.37). However, there is a small raise in mindset at the age interval ranging from 20–29 to 40–49, followed by a decrease among the older age groups. People with a growth mindset believe that human attributes are changeable, and that intelligence can develop through effort, practice, and education [60], and students’ beliefs and goals can powerfully influence their learning success [61]. In this context, although it is shown that grey matter tends to decrease with age and throughout life [62], it is linked to plasticity and to the process of maintaining and creating new neural networks due to practice and experience [63]. However, as the individual ages groups’ grey matter decreases, it may result in limitations in attainment of new knowledge and skills [3,31]. As a result, individuals might experience a decrease in growth mindset once they realize their abilities (both physically and cognitive) are not as adaptable as they used to be, and that learning new skills or knowledge may demand more effort and take a longer time. Such relationships might explain the decrease in growth mindset due to age. However, a limitation of this study concerns the fact that we do not use repeated measures. As different participants are measured in different ages, the results do not show actual individual development of the concepts across the life-span. Consequently, the significant differences between the younger age groups (13–19, 20–29, 30–39) and the oldest group (70–77) may indicate a change in learning and development approaches, which may be cultural. The younger generation may have been more exposed to information concerning intelligence change due to practice, through slogans such as “use it or lose it” and “use it and improve it” based on contemporary theories in neural development) [64,65,66,67]. As a result, younger people may have developed growth mindsets to a higher extent, compared to the older age groups. Furthermore, it is interesting to notice the curves for mindset as they are differing among males and females after the age group 30–39. Males’ mindsets increase on average until the age of 30–39, followed by a decrease until the age of 70–79, unlike in females, where mindset increases until the age 40–49 and decreases until the age of 70–79. In this context, one can notice the significant difference between the genders for the age group 60–69 years, which shows that females score significantly higher on growth mindset, compared to males. The significant difference (*p* = 0.023) between females and males for the whole sample is also interesting, underlining the importance of both age and gender associated to the development of mindset. According to Schlender et al. [55]), gender differences may exist because of their different socialization process, for example resulting in varying levels of academic mindsets. Additionally, the ability to adapt to age-related changes and losses, both physical and cognitive, is acted upon differently in male and females [68].

### 4.4. Limitations and Future Research

The current study has some limitations. As mentioned, repeated measures would have been more appropriate for the investigation of trait development, instead of a cross-sectional study. Based on this approach, the results may reveal generational differences, rather than development of the traits. Another limitation concerns the unbalanced number of participants in each age group, and a small sample size among the older age groups, which were more difficult to recruit. This could have caused non-significant results in patterns or trends we potentially could have discovered among the groups with smaller sample sizes. Furthermore, as this current study only focuses on a limited cultural context, i.e., Iceland and Norway, the generalizability of the findings may be reduced. Further studies should investigate if ethnic and socioeconomic factors relate to these variables. There is also a need to study these factors in relation to other achievement measures, as well as possible effects of interventions increasing these traits, across different age groups.

## 5. Conclusions

This study sought to explore the development of passion, grit, and mindset across life-span in relation to age and gender in a sample from 13 to 77 years. Generally, the patterns show that mindset and passion decrease across the life-span, while grit increases. The study finds gender differences (total sample) in passion (males score higher), and in mindset (females scores higher). These findings suggest that the three variables studied might be independent of each other, and partly related to age and gender. This knowledge might potentially be of relevance for future research exploring developmental patterns and associations between these factors. Broadening our knowledge about how passion, grit, and mindset fluctuate across the life-span could be of relevance for theoretical development within this area. The results can also reveal phases in life that may be more sensitive for interventions towards these qualities. The practical field should focus on the development of these concepts across the life-span, as they seem related to learning, achievement, and well-being. Gaining a better picture of these qualities can improve our understanding of how to enhance motivation and effort, as well as promote happiness and successful aging.

## Figures and Tables

**Table 1:** Mean scores for age, passion, grit, and mindset for age groups and gender.

		Age	Passion	Grit	Mindset
**Group**	***N***	Mean (SD)	**Mean (SD)**	Mean (SD)	Mean (SD)
**Total sample**	**1548**	**26.53 (17.77)**	**3.95 (0.65)**	**3.39 (0.60)**	**4.21 (0.99)**
Female	931	26.70 (11.81)	3.85 (0.66)	3.40 (0.59)	4.26 (0.93)
Male	617	26.27 (11.71)	4.09 (0.61) *	3.39 (0.60)	4.14 (0.06) **
**13**–**19 Group**	**242**	17.85 (1.47)	**4.11 (0.63)**	**3.47 (0.60)**	**4.22 (0.91)**
Female	140	18.30 (1.11)	3.95 (0.63)	3.41 (0.57)	4.20 (0.89)
Male	102	17.25 (1.68)	4.33 (0.56)	3.56 (0.63)	4.25 (0.93)
**20**–**29 Group**	**1014**	**22.68 (2.38)**	**3.95 (0.65)**	**3.32 (0.60)**	**4.18 (0.98)**
Female	606	22.46 (2.32)	3.86 (0.66)	3.34 (0.59)	4.20 (0.90)
Male	408	23.02 (2.43)	4.10 (0.60)	3.29 (0.61)	4.14 (1.08)
**30**–**39 Group**	**112**	**33.57 (2.79)**	**3.85 (0.63)**	**3.45 (0.56)**	**4.51 (1.01)**
Female	67	34.00 (2.86)	3.82 (0.67)	3.43 (0.60)	4.50 (0.98)
Male	45	32.93 (2.85)	3.89 (0.59)	3.47 (0.50)	4.51 (1.07)
**40**–**49 Group**	**62**	**44.39 (2.90)**	**3.89 (0.64)**	**3.58 (0.48)**	**4.66 (1.04)**
Female	43	43.72 (2.75)	3.89 (0.64)	3.58 (0.49)	4.83 (1.03)
Male	19	45.89 (2.73)	3.86 (0.65)	3.73 (0.44)	4.25 (0.98)
**50**–**59 Group**	**57**	**55.07 (2.68)**	**3.56 (0.68)**	**3.74 (0.55)**	**4.41 (0.99)**
Female	42	54.86 (2.72)	3.53 (0.69)	3.76 (0.58)	4.61 (0.92)
Male	15	55.67 (2.55)	3.64 (0.66)	3.71 (0.44)	3.84 (1.00)
**60**–**69 Group**	**38**	**63.29 (2.54)**	**3.82 (0.72)**	**3.56 (0.42)**	**3.96 (1.04)**
Female	20	62.95 (2.54)	3.78 (0.79)	3.58 (0.45)	4.42 (0.89)
Male	18	63.67 (2.54)	3.88 (0.65)	3.58 (0.45)	3.44 (0.98)
**70**–**79 Group**	**23**	**72.87 (2.46)**	**3.86 (0.71)**	**3.73 (0.51)**	**3.37 (0.84)**
Female	13	73.46 (2.60)	3.69 (0.83)	3.78 (0.58)	3.37 (0.87)
Male	10	72.10 (2.13)	3.95 (0.52)	3.67 (0.44)	3.37 (0.83)

**Group:** group as a whole; * significant difference between genders in favor of males (*t*-test, *p* < 0.001); ** significant difference between genders in favor of females (*t*-test, *p* = 0.02).

## Data Availability

The data that support the findings of this study are available from the corresponding author, [HS], upon reasonable request.
